# The use of arts‐based methodologies and methods with young people with complex psychosocial needs: A systematic narrative review

**DOI:** 10.1111/hex.13705

**Published:** 2023-01-11

**Authors:** Sally Nathan, Michael Hodgins, Jonathan Wirth, Jacqueline Ramirez, Natasha Walker, Patricia Cullen

**Affiliations:** ^1^ School of Population Health UNSW Sydney Sydney New South Wales Australia; ^2^ School of Clinical Medicine UNSW Sydney Sydney New South Wales Australia; ^3^ The George Institute for Global Health UNSW Sydney, New South Wales Australia Newtown New South Wales Australia; ^4^ Ngarruwan Ngadju: First Peoples Health and Wellbeing Research Centre University of Wollongong Wollongong New South Wales Australia

**Keywords:** arts‐based, complex needs, psychosocial, qualitative, young people

## Abstract

**Background:**

Arts‐based methodologies and methods (ABM) can elicit rich and meaningful data with seldom‐heard groups and empower participants in research. Young people with complex psychosocial needs could be better engaged in research using arts‐based approaches to overcome communication and literacy issues as well as distrust of those with power, including researchers. A critical review of the use and impact of ABM among this population is timely. The purpose of this review is to synthesize and examine the experience and use of ABM with young people with complex psychosocial needs.

**Methods:**

A systematic narrative literature review was conducted with a search of the literature from 2009 to 2021. All abstracts were reviewed independently by two authors and full papers were screened for eligibility against inclusion and exclusion criteria. Data synthesis focused on a descriptive numerical summary and a thematic analysis focused on key patterns across papers relating to the review objectives.

**Results and Discussion:**

A total of 25 papers were included. The most common issues of focus were mental health (*n* = 10) and homelessness (*n* = 11) and methods using Photovoice (*n* = 12) and Body Mapping (*n* = 5). Individual interview data (*n* = 20) were the most commonly analysed, followed by created works (*n* = 19). Less than half the studies involved young people in the interpretation of the data collected. Knowledge translation was not described in almost half the studies, with public exhibits (*n* = 7) and forums with service providers (*n* = 4) being the most common activities. Key themes across the studies were valued over traditional methods in eliciting data, ABM as an approach to engage these young people in research and the impact of the use of ABM on participants and on key stakeholders through knowledge translation.

**Conclusions:**

The growing field of ABM presents opportunities to enhance research with young people with complex psychosocial needs by promoting meaningful exploration of experiences, engaging participants in research and strengthening knowledge translation. The involvement of young people in the interpretation of data and ensuring that knowledge translation occurs are key areas for future attention.

**Patient or Public Contribution:**

The findings of this review will inform future research to improve the engagement of young people with complex psychosocial needs in research and promote power sharing between researchers and research participants.

## 1 BACKGROUND

Arts‐based research methodologies and methods (ABM) are receiving increasing attention in health and social sciences because of their potential to elicit deeper, richer, more meaningful data from participants' perspective.^1^ Drawing on narrative, visual, audio and experiential forms of artmaking, arts‐based research can elucidate otherwise hidden knowledge as participants give meaning to their experiences in forms beyond the spoken or written word.^2^, ^3^, ^4^, ^5^ ABM can involve the integration of artmaking in the data collection process, often in conjunction with other more traditional data collection methods such as interviews, focus groups or ethnographic research. ABM can also be used in the research process to create, disseminate or translate research, often through exhibitions, installation or performance.^1^ For the purposes of this review, ABM are understood as those in which participants engage in a creative practice of self‐expression at any point in the research process. Practices of creative self‐expression might include the use of art forms such as poetry, drawing, mapping, collage, photographs/photovoice, participatory video, digital storytelling (DST), drama and image theatre or theatre.^6^, ^7^ We define methodologies as the assumptions and principles in a particular research approach and methods as the research action, that is, the techniques for gathering data or evidence.^8^ We understand methods as the tools, techniques or procedures used to gather the evidence.^8^ In past reviews, authors have noted that methodology and method have been used interchangeably in the arts‐based research contexts,^1^ hence why we have included both terms.

ABM have been used in a range of settings and with different participant groups.^3^, ^9^, ^10^, ^11^ With a focus on the dissemination of knowledge in arts‐based health research, Fraser and al Sayah examined what methods are considered as arts‐based research and how these are used in health where studies involved children and adults.^11^ Photographs and drawings were found to be the main methods used and studies aimed to produce new knowledge or translate knowledge to practice in the varying contexts of HIV, heart disease and life‐threatening conditions.^11^, p.127 Leavy^12^ asserts that ABM can complement or even improve upon traditional qualitative research methods, as it brings meaning‐making to the forefront of the process of data collection and creation, enriching understanding and empowering participants through the participatory nature of art production. ABM are frequently grounded in participatory methodologies that aim to empower participants as partners in research.^11^ Empowerment can mean participant control over art‐making decisions, data selection or data analysis and other areas of the research process including guiding research to explore areas and themes relevant to their experience.^11^ ABM can therefore promote more meaningful dialogue than more traditional qualitative methods and support discussions around complex or sensitive issues.^11^, ^12^ ABM have also been shown to produce data that are viewed as relatable, informative and impactful by both expert and wider audiences, priming them for use in knowledge translation through exhibitions, installation or performance.^1^, ^11^, ^12^ The emotive capacity of art can promote audiences into thinking differently, deeply and empathetically about others' experiences.^12^

While ABM have been used to good effect for conducting research with some marginalized population groups, there has been minimal exploration of the benefits and barriers to these methodologies and methods with young people with mental health and related issues.^1^ D'Amico et al.^7^ discuss the use of arts‐based, visual and digital methods and ‘their potential to enhance the quality of data collected and to engage and empower child and youth participants’ (p. 529) facing global adversity. De Vecchi et al.^13^ note in their scoping review the ‘lack of uptake’ (p. 191) of digital storytelling in mental health research despite ‘implications for the development of recovery‐oriented mental health services’ (p. 191). Challenges to the use of ABM are time and resource constraints.^14^ The provision of tools, time and support for the creation of art projects can be expensive and may not scale to larger sample sizes.^15^ Ethical issues, such as ownership, anonymity and risks of retraumatization, are other challenges to the use of art in research.^11^, ^16^

A critical review of the use and impact of ABM among young people with complex psychosocial needs is timely. We understand complex psychosocial needs as multiple levels of need, for example, a diagnosed mental health issue, but also including issues such as unstable housing, involvement with the social care and justice systems and co‐occurring drug and alcohol issues.^15^, ^17^, ^18^, ^19^, ^20^, ^21^, ^22^, ^23^, ^24^, ^25^ This definition and the scope of included studies in the current review are addressed in further detail in the methods section. There are many challenges in research, including the use of arts‐based methodologies with young people with complex needs, particularly those with poor literacy and high levels of distrust of those with power, including researchers.^14^, ^26^ The rapidly changing field of social media and technology use among young people also underscores the need to assess the use of other technologies beyond the traditional photovoice, photo elicitation and other visual storytelling devices used in past research with young people.^14^ Ostensibly, these methodologies and methods are appropriate for this population because they sit within an emancipatory research paradigm, emphasizing empowerment and knowledge creation within the process of the research rather than merely the end‐product.^27^, ^28^

Arts‐based methodologies are thought to extend traditional research knowledge boundaries to value and include participants voices and to seek to provide participants with greater power in the research process.^16^ This is because artmaking in the research process creates space for participants to safely control the expression of their perspectives. This has been powerfully demonstrated for participants who are often marginalized or silenced within society.^29^, ^30^ Often, participants' expressions lead to a ‘deeper understanding’ (p. 683) of the way people experience their health and well‐being by not only engaging participants in the research process but also by motivating policymakers and healthcare professionals into action to improve services.^16^ Published studies highlight several beneficial outcomes from using arts‐based, visual and digital methods in research such as their ability to provide counter‐narratives and reduce stigma. De Jager et al.^31^ found that studies using Body Mapping methods empowered participants by enabling them to produce a first‐person nonverbal narrative. The method was found to engage young participants and balance power dynamics between the competing world views of participant and researcher. In their systematic review of digital storytelling in research, de Jager et al.^3^ note therapeutic benefits for participants and potential to reach policymakers and create positive social change.

This systematic review is unique, as previous systematic reviews have tended to focus on the use of a specific arts‐based, visual and digital research methodologies, while we are instead focussing on a particular population subgroup: young people with complex psychosocial needs and the use of any arts‐based methodology or method to engage them in research. More specifically, the objectives of the review were to identify and synthesize the evidence about

(1) The types of arts‐based research methodologies and methods used in research to measure experience; access to and/or experience of health and related social service and impact or outcomes of services among young people with complex psychosocial needs.
(2) How arts‐based methods and/or creative outputs were used at different research stages.
(3) Reported improvements in data elicitation about access, experience and outcomes of health and related social services compared to more ‘traditional’ qualitative approaches.
(4) The involvement of young people in meaning‐making.

Synthesis of the evidence about the methodologies and methods used, their impact on data collected, policy and practice and young people themselves will inform thinking around new innovations, use and benefits of arts‐based visual and digital research methodologies and methods to improve policy and health programmes for young people with complex psychosocial needs.

## 2 METHODS

The systematic review was conducted in accordance with PRISMA guidelines^32^ and has been registered with PROSPERO (Examining the use of arts‐based, visual and digital methods in research with vulnerable youth, registration number CRD42020161675).

### 2.1 Search strategy and information sources

Four electronic databases were systematically searched: Medline, ProQuest, Embase and CINAHL. A comprehensive set of search strategies was implemented to identify all relevant studies (see Supporting Information: 1). Pilot searches were run to test the terms using key articles expected to meet our inclusion criteria and minor changes were made to search terms to ensure that key papers were included.

### 2.2 Inclusion criteria

Studies included were those using ABM, either on their own or in combination with or other qualitative or quantitative research methods, with young people aged between 10 and 24 years with complex psychosocial needs (e.g., have directly experienced mental health difficulties that would meet criteria outlined in the Diagnostic and Statistical Manual of Mental Disorders or have experiences of homelessness in the community). Young people who are homeless are a deliberate focus of this review, given that the incidence of mental health disorders in this group is significantly higher than among young people in the general community and they are less likely to have a diagnosis.^23^, ^24^ Those studies that were focused on young people with a diagnosed learning and developmental disorder or intellectual disability were not included after discussion by the full research team. It was agreed that they constituted a group of participants for whom arts‐based approaches would be valuable, but that ABM would require substantial tailoring specifically to their needs, which warranted a separate and more focused systematic review. Detailed inclusion and exclusion criteria are provided in Supporting Information: 2.

### 2.3 Review process

**Figure 1:**
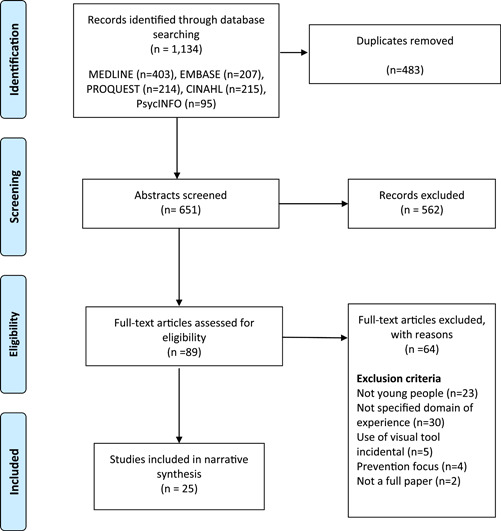
Study selection process

Articles identified were exported from each individual database using export tools in EndNotex9 bibliographic software and duplicates were removed.^33^ At least two independent reviewers from the research team were involved in the processes of screening for eligibility, extraction and data synthesis. At full‐text review, the reasons for exclusion were recorded. The results of the search in the Preferred Reporting Items for Systematic Reviews and Meta‐analyses (PRISMA) flow diagram^32^ are provided in Figure 1.

### 2.4 Data extraction

A data extraction tool was developed and piloted by the research team. The data extracted included details about the population, setting, domain of experience, study methodology and methods, data collected and analysed (including young people's involvement) and knowledge translation strategies. The data extraction tool with an example is provided in Supporting Information: 3.

### 2.5 Data synthesis

The synthesis first focused on a descriptive numerical summary of countries, the papers that focused on health issues, methodology, arts‐based method(s) used, data collected and analysed, approach to analysis and knowledge translation strategies. Thematic analysis was then used to identify patterns across articles regarding how ABM are used in research with young people with complex psychosocial needs, focusing on the value of ABM, the involvement and impact on study participants and challenges.^34^ To achieve this analysis, two reviewers:

(1) Familiarized themselves with the studies and findings in each of the papers referring to the extraction table and full papers as required;
(2) Generated an initial coding scheme to organize data thematically discussed with the full research team;
(3) Amalgamated/organized codes to generate potential theme and
(4) Reviewed themes in terms of their salience across the reviewed articles and in relation to the review objectives.

### 2.6 Assessment of methodological quality

This systematic review was concentrated on the research process and approach, methods used, data obtained, impact on participants and knowledge translation rather than overall study quality as the focus of systematic reviews is to assess evidence of interventions on health outcomes. However, data were extracted in relation to evidence about the value of ABM in improving data quality, impacts of the research on service delivery or policy and the impacts on study participants, for example, whether ABM enhance the quality of data in terms of richness, recruitment, retention and engagement of participants, which are key challenges in research on sensitive issues and with ‘vulnerable’ participants.^35^

## 3 RESULTS

### 3.1 Descriptive results

ABM were used with young people with complex psychosocial needs in studies across several diverse countries and continents. Most studies were carried out in the United States (*n* = 6) and Canada (*n* = 5), followed by Australia (*n* = 4), Central/South America (*n* = 4), Africa (*n* = 2) and one each in South Korea, the United Kingdom, France and Sweden.

#### 3.1.1 Domains

Issues or conditions most often studied were mental health (*n* = 10), homelessness (*n* = 11), general complex support needs (*n* = 2) and addiction (*n* = 2).

#### 3.1.2 Methodological frameworks

Participatory methodologies (*n* = 10) were the most common, followed by interpretative phenomenological and hermeneutic (*n* = 6), unspecified qualitative methodology (*n* = 7), grounded theory (*n* = 1) and narrative approaches (*n* = 1).

#### 3.1.3 Arts‐based methods

The definitions of arts‐based methods and exemplar papers from this review are provided in Table 1. Photovoice (*n* = 12) was the most commonly used method, followed by Body Mapping (*n* = 5). These findings were consistent with a systematic review conducted by Fraser and al Sayah,^11^ who found that visual methods, such as photos and Body Mapping, were the most used ABM.^2^

**Table 1:** Definitions of arts‐based methods and use in included studies

Arts‐based method	Definition	*n* (%)	Paper examples
Photovoice (including Photo‐blogging/participatory photography)	Photovoice is an established method developed initially by health promotion researchers Wang and Burris.^64^ The method involves participants taking photos guided by a research question, which are then used to help them reflect upon and explore the reasons, emotions and experiences that have guided their chosen images.	15 (58%)	The study of Bender et al.,^54^ ‘Asking for Change’ Photovoice intervention with young people who were homeless aimed to (1) build relationships and connection, (2) teach social, emotional, leadership and photography skills and (3) empower young people to be social change agents. Guided by a professional photographer, young people were each provided with an electronic tablet to take photos about their lives to share and discuss with the group. A mixed‐methods evaluation showed that the approach was feasible and highly acceptable, created new opportunities to connect and was associated with improvements in communication skills, social connectedness, resiliency and well‐being.
Photo or visual‐elicitation	Photo and visual elicitation methods involve using a photograph or other visual stimulus as a support during a research interview.^65^	3 (11%)	The study of Lecomte et al.^37^ aimed to understand the role of food in family interactions amongst teenagers with Bulimia Nervosa. As part of their method, participating teenagers were asked to take a photograph of the table after a family meal before clearing to discuss with the researcher during an interview. The selected photograph was displayed on a computer screen during the interviews and was the basis for the questions asked. The same picture was also used for an interview with their parents.
Body Mapping	Body Mapping involves creating life‐sized artworks of the human body to visually depict an individual's perception of their body, identity and experiences. Most commonly, the method involves drawing an outline in pairs of a person's body in a position they wish to be represented on a large piece of drawing paper and then using a range of art materials to fill in the Body Map in response to questions posed in a workshop.^31^	5 (19%)	The study of Macken et al.^2^ aimed to explore the use of Body Mapping as a research method with young people in residential treatment for drug and alcohol issues and to examine how Body Mapping can engage young people in exploring their strengths and sources of support during treatment. They found that Body Mapping produced richer data and accessed new narratives not usually captured in more traditional interviews, in a subsequent interview where young people explained their Body Map.
Digital storytelling	Digital storytelling (DST) involves using multimedia consisting of images/segments of video with background music and voiceover narrative to explore experiences.^66^	1 (4%)	The study of Boydell et al.^56^ involved young people diagnosed with psychosis. Participants attended a DST workshop session to produce individual digital stories describing how they manage psychosis in everyday life. Overall, young people found the story creation process both challenging and rewarding. For instance, although creating the story was emotionally difficult and ‘scary’ for participants, they reported feeling better and a sense of relief when it was completed. Participants found it difficult to make decisions about what part of their story to tell.
Filmmaking	Filmmaking was involved in only one included study, which involved co‐production of a film to disseminate research findings.	1 (4%)	The study of Dunn et al.^52^ details their efforts to find ways to provide information about depression and help‐seeking beyond traditional academic audiences, specifically to other young people experiencing depression. This involved the creation of a film through a collaborative workshop where young people shared their experiences, decided on the tone, tenor and message of the film, identified their primary audience and produced the bulk of the audio and visual material.
Song writing	Song writing was a method used in only one included study, which involved co‐writing a song to analyse and summarize data generated from qualitative methods.	1 (4%)	The paper of Fairchild and McFerran^55^ described a music workshop that involved collaborative song writing with children experiencing homelessness and family violence. This included engaging participants in creative and child‐centred forms of data generation, analysis and presentation of the findings. The researchers focused on the use of group song writing to co‐create knowledge and understanding and to ensure that the final product represented the young people in ways that they could recognize and resonate with.

#### 3.1.4 Data collected and analysed

Table 2 shows the types of data used in analysis in these studies. Individual interview data (*n* = 20) were the most common data type, followed by created works (*n* = 19), field notes (*n* = 8) and group interview data (*n* = 7). Many studies used more than one type of data for analysis and often used created works as prompts and devices to explore issues during interviews.

**Table 2:** Use of arts‐based methods and/or creative outputs at different research stages (*n* = 25)

Research stage	*n* (%)
Data collection:	
All studies involved young people's participation in the creation of art or image	25 (100)
Data used in analysis:	
Artwork and associated descriptors^36^, ^38^, ^39^, ^40^, ^41^, ^43^, ^44^, ^45^, ^46^, ^48^, ^49^, ^50^, ^51^, ^52^, ^53^, ^54^, ^56^, ^57^, ^59^, ^60^	19 (76)
Individual interviews^36^, ^37^, ^38^, ^39^, ^40^, ^42^, ^43^, ^44^, ^46^, ^47^, ^48^, ^49^, ^50^, ^53^, ^54^, ^55^, ^56^, ^57^, ^58^, ^59^	20 (80)
Group interviews or discussions^36^, ^38^, ^45^, ^49^, ^51^, ^52^, ^57^	7 (28)
Researcher field notes^40^, ^45^, ^46^, ^49^, ^50^, ^55^, ^57^, ^58^	8 (32)
Quantitative self‐report measures^44^, ^55^	2 (8)
Other^40^, ^44^, ^55^, ^60^	4 (16)
Knowledge translation:	
Not described^37^, ^39^, ^40^, ^42^, ^45^, ^48^, ^49^, ^50^, ^56^, ^57^, ^58^, ^59^	12 (48)
Forums with service providers^38^, ^41^, ^44^, ^46^	4 (16)
Exhibition of artworks^36^, ^44^, ^51^, ^52^, ^53^, ^55^, ^60^	7 (28)
Other^38^, ^43^, ^60^	3 (12)

#### 3.1.5 Approach to analysis

Thematic analysis (*n* = 9) was the most common approach to analysis, followed by unspecified qualitative analysis (*n* = 8), content analysis (*n* = 4) and visual narrative analysis (*n* = 2). In our review, almost a third of the studies did not report any particular theoretical orientation in their analysis. In their systematic review of ABM, Fraser and al Sayah identified a need for a more explicit discussion of theoretical underpinnings in this area of research.^11^

#### 3.1.6 Knowledge translation

Knowledge translation, when described, occurred through public exhibits (*n* = 7) and forums with service providers (*n* = 4). In nearly half the studies, knowledge translation activities were not described. See Table 2.

### 3.2 Thematic analysis results

#### 3.2.1 Enriching data through arts‐based research methods

Almost all studies emphasized that the use of ABM elicited richer data than solely relying on traditional qualitative methods. Several studies reported the potential of arts‐based research methods to elicit information that is not readily expressed in words or text.^36^, ^37^, ^38^, ^39^, ^40^ Several authors argued that ABM allowed for participants' viewpoints to be represented with greater authenticity and depth.^2^, ^37^, ^40^, ^41^, ^42^, ^43^, ^44^ Young people were also reported to be able to better disclose and share complex and difficult experiences and emotions.^2^, ^36^, ^44^, ^45^, ^46^, ^47^

#### 3.2.2 Engaging young people with complex psychosocial needs

ABM were found to be highly engaging for young people who are traditionally hard to reach and retain in research.^36^, ^41^, ^45^, ^48^, ^49^, ^50^ Several authors noted its utility in initiating and facilitating dialogues with young people, particularly about difficult and confronting issues.^2^, ^37^, ^42^, ^46^, ^51^, ^52^ Studies also described the use of created art as prompts to initiate an interview, generate deeper exploration or at the end of interviews as a means of keeping young people engaged.^37^, ^51^ This finding was consistent with Bagnoli's review of ABM, in which they found ABM to be useful as an ‘ice breaker’.^53^ ABM were also reported to be useful in holding young peoples' attention, particularly through research elements requiring concerted participation.^2^, ^43^ The research process was reported to be enjoyable and valuable by participants who found the use of art to be more familiar and comfortable than the spoken word.^2^, ^42^, ^45^, ^49^, ^52^, ^54^

#### 3.2.3 Sharing power

Participation is considered central to ABM, as collaboration with researchers empowers participants and allows a richer representation of experience. We were therefore interested in how often ABM allowed for substantive participatory involvement in analysis. In the studies reviewed, there were several forms of participation in analysis: identification of themes or topics (*n* = 8), co‐analysis by guided group discussion (*n* = 7), selected pictures for analysis (*n* = 4) and analysis through art production (writing lyrics, etc.) (*n* = 2). In our review, 12 studies (44%) involved some form of participation in analysis. Several authors noted that ABM promoted a more democratic process in research and valued young people as partners and experts^2^, ^38^, ^42^, ^47^, ^49^, ^51^, ^52^, ^54^ and promoted a reciprocal empathetic relationship between researchers and young people.^2^, ^38^, ^43^ Due to the creative nature of ABM, the skills and assets of young people were seen to be more valued.^42^, ^52^ Young people were also reported by the authors to be more able to influence the direction of research through participation than in research using more traditional methods.^36^, ^48^, ^49^

#### 3.2.4 Impact on participants and therapeutic effect

In several studies, authors noted that ABM improved young peoples' self‐efficacy or self‐worth.^36^, ^54^, ^55^, ^56^ The collaborative nature of ABM was reported to facilitate the development of participants' communication and social skills.^45^, ^48^, ^52^, ^54^, ^57^ In some studies, participants were able to share common experiences with peers and developed new support networks.^43^, ^45^, ^46^, ^54^ Young people were reported to have gained new creative skills and took pride in the creation of art they found to be meaningful.^45^, ^48^, ^52^, ^56^ ABM also served as an emotional outlet for young people and promoted self‐reflection, offering them an opportunity to discover unexpected strengths, resiliency, optimism and process their past experiences.^2^, ^36^, ^38^, ^41^, ^45^, ^47^, ^48^, ^49^, ^50^, ^51^, ^54^, ^56^, ^57^, ^58^, ^59^ In one study, ABM was noted as having a calming effect.^2^ Bender et al.,^54^ who used a validated self‐report measure, found that ABM improved young peoples' social connectedness, resiliency and personal well‐being.

#### 3.2.5 Challenges in the use of arts‐based research methods

The most often cited challenge to implementing ABM was that it was resource‐ and time‐intensive.^41^, ^52^, ^54^, ^56^ Ethical issues, such as ownership over art, consent and the presentation of photographs including minors, were also reported.^45^, ^47^, ^48^, ^50^, ^54^, ^59^ Issues pertaining to group interactions, participant attention and retention arose due to the creative and relatively unstructured nature of the research, as well as the complex psychosocial needs of the participants in a few studies.^51^, ^55^ Some researchers expressed a concern that ABM may expose young people to retraumatization by triggering past experiences^50^, ^52^ and highlight the need for support to be provided during and following ABM projects.

## 4 DISCUSSION

Several key themes emerged from our analysis and synthesis of the use of ABM across a diverse range of studies with young people with complex psychosocial needs. ABM's value over traditional methods, the engagement of young people in research and the impact on participants and on others through knowledge translation were key areas of focus in this systematic review.

In almost all studies, ABM were found to elicit rich and insightful data from young people with complex psychosocial needs. This is an important finding, as research with these young people can be challenging, including in terms of data quality.^60^ A major barrier to productive dialogue with young people in research has been found to result from their discomfort and hesitancy to express themselves through words.^14^, ^26^ ABM bypasses this challenge, effectively capturing nonverbal and preconscious information.^2^, ^36^, ^37^, ^38^, ^40^, ^61^ For example, Willis et al.^39^ found that ABM were effective in ‘externalizing somatic and emotional experiences’ (p. 2). Importantly, ABM also enabled young people to identify strengths and positive traits,^43^ which are often overshadowed by a more deficit discourse in more conventional approaches.^1^, ^2^, ^10^ A focus on resilience and optimism in research can also translate to interactions outside of the research process as found by Macken et al.^2^ in their study, in which young people took their body maps to counselling sessions to explore their strengths and sources of support. While most studies in this review reported positive psycho‐social outcomes from participation in the research, some studies noted a concern regarding retraumatization.^50^, ^52^ Attention to ensuring appropriate psychological support is important in future studies.

Qualitative research methods are argued to be more effective if they can engage participants and therefore collect data that better reflect the experiences and meaning‐making of the participants.^62^ In this review, ABM was found to be highly engaging for young people and promoted interaction with researchers and in the research process.^2^, ^36^, ^37^, ^41^, ^42^, ^43^, ^48^, ^49^, ^50^, ^51^ By holding young peoples' interest and curiosity, the use of art allowed for a ‘progression from factual, descriptive questions to more personal ones’ (p. 3).^36^ The use of art appeared to foster an openness to self‐expression and communication that allowed for more involved, sustained and enjoyable participation in the data collection process and shifted the unequal power dynamics often inherent in research.^2^, ^14^, ^42^, ^49^, ^52^, ^54^

ABM are often grounded in a participatory methodology ^14^ as we found was used in 10 of the 25 included studies. Leavy^12^ asserts that in ABM, participants are often viewed and valued as equal collaborators, shifting the traditional researcher‐researched hierarchy. As there is no correct way of understanding art, ABM democratizes meaning and decentralizes power between researchers and young people.^12^ From this position, it is argued that compassionate and productive reciprocal relationships can be formed that lessen the barriers of age and authority.^38^, ^52^ As a result of equalizing power differentials, young people can more readily engage with research and share their experience.^3^, ^50^ Several studies in this review noted the importance of participation in ABM to promote meaningful engagement and therefore more authentic data.^38^, ^49^, ^50^ Importantly, empowering young people with a voice and a platform can actively address the stigma and inequity and underlying power differentials that they may experience both in research and outside of research.^38^, ^45^, ^48^, ^52^ In nonparticipatory methodologies, participants can be detached from the analysis and meaning‐making about the data they produced. In contrast, when young people were involved in the identification and development of themes, as was the case in almost half of the studies, researchers reported greater confidence that the findings produced reflected participants' lived experience.^14^, ^63^

ABM can have a profound effect on participants. Young people reported being empowered through the acquisition of creative skills and the production of art.^48^ An opportunity to create something meaningful can enable young people with complex psychosocial needs to build their sense of self, identity and self‐esteem and change their and others' perceptions of what they can accomplish.^45^, ^48^, ^52^, ^56^ ABM can also have direct therapeutic benefits.^3^ In our review, ABM appeared to draw out young peoples' strengths, resilience and optimism.^36^, ^38^, ^41^, ^45^, ^47^, ^48^, ^49^, ^54^, ^56^, ^57^, ^58^, ^63^ However, Forge et al.^51^ expressed concern that participant retention was impacted by significant barriers in the lives of the young people, with only two studies reporting making participation in the research flexible to account for these barriers.^42^, ^52^

While the use of art in research has been found to increase the accessibility of findings to the general public, key questions remain about how ABM can facilitate knowledge translation and influence policy and practice.^1^, ^12^ In our review, 12 studies (48%) did not describe knowledge translation activities. The lack of attention to knowledge translation in almost half of the reviewed studies was consistent with the findings of de Jager et al.^3^ and highlight an area that requires attention in future studies to maximize the impact of the use of ABM. While ABM can have important positive impacts in the research setting, it is vital that meaningful and tangible changes are focussed on service provision and policy.^12^ A common theme throughout many of the reviewed studies was young peoples' strong desire to share their stories and advocate for issues through their art, underscoring the importance of knowledge translation activities.

## 5 FUTURE RESEARCH

Future research should explore any differences in the feasibility and acceptability of different methods and their benefits for young people with distinct issues, such as those experiencing mental health issues compared to those who are homeless and also for those with different gender identities and diverse ways of thinking, learning, processing and behaving, often referred to as neurodiverse individuals. The issue of retraumatization or the triggering of past difficult experiences was raised in some studies. Future research using ABM should identify and evaluate approaches to keeping participants safe and the types of supports that are provided to mitigate any harms.

## 6 CONCLUSION

ABM is a growing field, demonstrated by having 22 of the 25 papers identified and included that were published in the last 5 years (since 2017). The use of ABM with young people with complex psychosocial needs was reported to elicit rich and authentic data, while engaging participants and empowering them as partners in research. ABM was reported as adding substantial utility to more traditional qualitative research approaches including promoting meaningful exploration of participant experience. ABM was found to have benefits for young people, who gained creative skills, social networks and an opportunity for self‐reflection. Knowledge translation is a key area for future attention, given the potential of the data generated through ABM to impact on policy and practice.

## CONFLICT OF INTEREST

The authors declare no conflict of interest.

## Supporting information

Supporting information.

Supporting information.

Supporting information.

## Data Availability

The author has provided the required data availability statement, and if applicable, included functional and accurate links to said data therein.
