# Supplementary material for: The use of arts‐based methodologies and methods with young people with complex psychosocial needs: A systematic narrative review
Source: Health Expect. 2023 Jan 11;26(2):795–805. doi: 10.1111/hex.13705 (PMC10010092; doi:10.1111/hex.13705)
Supplement: Supplementary file 1 — Supporting information. [file HEX-26--s003.docx]

**Supplementary Materials 1:** Search Strategy

Limited to 2010-2021, English language

(teen* OR adolescen* OR youth OR "young people" OR "young adult*")

AND

((digital OR digitally OR visual*) NEAR/4 (story* OR stories OR narrat* OR dialogue*)) OR (video* NEAR/1 (stories OR story* OR diar* OR interview*) OR ("body map*" OR photovoice OR "photo voice" OR "photo elicitation") OR ("arts based" OR "arts informed" OR visual) NEAR/3 (participatory OR ethnographic* OR research))

AND

((mental OR psych*) NEAR/1 (health OR disorder OR sickness OR illness)) OR ((alcohol* OR substance OR drug* OR narcotic* OR chemical) NEAR/4 ("use" OR abuse OR addiction OR habit OR depend* OR treat* OR rehab*)) OR (homeless* OR displace* OR transien* OR destitute OR migrat* OR street*) OR ((social OR community OR support) NEAR/1 (services OR welfare OR relief))

AND

stype.exact("Scholarly Journals")

AND

la.exact("English")
