# Supplementary material for: The use of arts‐based methodologies and methods with young people with complex psychosocial needs: A systematic narrative review
Source: Health Expect. 2023 Jan 11;26(2):795–805. doi: 10.1111/hex.13705 (PMC10010092; doi:10.1111/hex.13705)
Supplement: Supplementary file 2 — Supporting information. [file HEX-26--s002.docx]

**Supplementary Materials 2** Inclusion and Exclusion Criteria

| Inclusion Criteria |
| --- |
| 1. Published studies that (have developed) or used visual, digital or arts-based methods to collect data to measure:    1. Experience of a mental health condition (any disorder in DSM which includes addiction) and/or experience of homelessness (in community), or    2. access to and/or experience of a health or social service for a. above, or    3. the impact or outcomes of a health or social service for a. above |
| 1. Published systematic reviews of studies that have developed or used visual, digital or arts-based methods to collect data to measure experience, or the impact or outcomes of health or social services for a. above |
| 1. Studies published in English, as a journal article |
| 1. Published since 2010 |

| Exclusion Criteria |
| --- |
| 1. The study population are not young people themselves. |
| 1. Program and/or development and trial of measurement method or tool focussed on a specific physical disease only. |
| 1. Program incidentally uses a visual, digital or arts-based method (s) or tool(s) for measurement, but it is not described in sufficient detail to answer any of the review questions or does not use any visual, digital or arts-based method (s) or tool(s). |
| 1. The method or tool is used for therapeutic benefits or as a clinical tool only not for data collection in a research study. |
| 1. Study focussed on collecting data about prevention. |
| 1. Thesis or dissertation, or book chapter or conference abstract. |
| 1. Research participant are young people, but they have not directly experienced a. above. |
| 1. Research participants diagnosed with a developmental disorder or intellectual disability. |
| 1. Focus on experience of migration. |
