# Supplementary material for: The use of arts‐based methodologies and methods with young people with complex psychosocial needs: A systematic narrative review
Source: Health Expect. 2023 Jan 11;26(2):795–805. doi: 10.1111/hex.13705 (PMC10010092; doi:10.1111/hex.13705)
Supplement: Supplementary file 3 — Supporting information. [file HEX-26--s001.docx]

**Supplementary materials 3** Extraction Table

| Author | Domain | Participants, setting/country | Study Aim(s) | Methodology, methods, analysis | Participant engagement and experience | Knowledge Translation | Challenges |
| --- | --- | --- | --- | --- | --- | --- | --- |
| EXAMPEL PAPER Fortin et al.  (2015) | Homelessness | n=5 (5F)  Age: 18-24  Community  Canada | To understand how young mothers experience homelessness.  To improve the appropriateness and sensitivity of formal and informal supports. | Participatory Methodology  *Method:* Photo-blogging Workshop  *Data:* Photographs; Blog text.  *Analysis:* Co-analysis - Meanings discussed in workshops to identify themes. | Allowed for collective dialogue;  Facilitated self-reflection. | Presenting findings to service providers. | Resource intensive;  Not all participated in analysis workshops. |
